# Social Determinants of Health Data Improve the Prediction of Cardiac Outcomes in Females with Breast Cancer

**DOI:** 10.3390/cancers15184630

**Published:** 2023-09-19

**Authors:** Nickolas Stabellini, Jennifer Cullen, Justin X. Moore, Susan Dent, Arnethea L. Sutton, John Shanahan, Alberto J. Montero, Avirup Guha

**Affiliations:** 1 Case Western Reserve University School of Medicine, Case Western Reserve University, Cleveland, OH 44106, USA; 2 Department of Hematology-Oncology, University Hospitals Seidman Cancer Center, Cleveland, OH 44106, USA; 3 Faculdade Israelita de Ciências da Saúde Albert Einstein, Hospital Israelita Albert Einstein, São Paulo 05652-900, SP, Brazil; 4 Department of Medicine, Medical College of Georgia, Augusta University, Augusta, GA 30912, USA; aguha@augusta.edu; 5 Case Comprehensive Cancer Center, Cleveland, OH 44106, USA; 6 Center for Health Equity Transformation, Department of Behavioral Science, Department of Internal Medicine, Markey Cancer Center, University of Kentucky College of Medicine, Lexington, KY 40506, USA; 7 Duke Cancer Institute, Duke University, Durham, NC 27708, USA; 8 Department of Kinesiology and Health Sciences, College of Humanities and Sciences, Virginia Commonwealth University, Richmond, VA 23284, USA; 9 Cancer Informatics, Seidman Cancer Center, University Hospitals of Cleveland, Cleveland, OH 44106, USA; 10 Cardio-Oncology Program, Medical College of Georgia, Augusta University, Augusta, GA 30912, USA

**Keywords:** breast cancer, cardiovascular disease, machine learning, social determinants of health, race, disparities, prediction, cardiooncology

## Abstract

**Simple Summary:**

This research aimed to investigate if adding social determinants of health (SDOH) to predictive models improves major adverse cardiovascular events (MACE) predictions in breast cancer patients, as cardiovascular disease is their leading cause of death. ML models, incorporating SDOH, demographics, risk factors, tumor characteristics, and treatments, were developed and compared. The results showed that including SDOH enhanced ML model performance in forecasting MACEs within two years of breast cancer diagnosis, especially for non-Hispanic Black patients. These findings offer more accurate risk assessments and personalized care insights for breast cancer patients, while also guiding efforts toward achieving healthcare equity.

**Abstract:**

Cardiovascular disease is the leading cause of mortality among breast cancer (BC) patients aged 50 and above. Machine Learning (ML) models are increasingly utilized as prediction tools, and recent evidence suggests that incorporating social determinants of health (SDOH) data can enhance its performance. This study included females ≥ 18 years diagnosed with BC at any stage. The outcomes were the diagnosis and time-to-event of major adverse cardiovascular events (MACEs) within two years following a cancer diagnosis. Covariates encompassed demographics, risk factors, individual and neighborhood-level SDOH, tumor characteristics, and BC treatment. Race-specific and race-agnostic Extreme Gradient Boosting ML models with and without SDOH data were developed and compared based on their C-index. Among 4309 patients, 11.4% experienced a 2-year MACE. The race-agnostic models exhibited a C-index of 0.78 (95% CI 0.76–0.79) and 0.81 (95% CI 0.80–0.82) without and with SDOH data, respectively. In non-Hispanic Black women (NHB; *n* = 765), models without and with SDOH data achieved a C-index of 0.74 (95% CI 0.72–0.76) and 0.75 (95% CI 0.73–0.78), respectively. Among non-Hispanic White women (*n* = 3321), models without and with SDOH data yielded a C-index of 0.79 (95% CI 0.77–0.80) and 0.79 (95% CI 0.77–0.80), respectively. In summary, including SDOH data improves the predictive performance of ML models in forecasting 2-year MACE among BC females, particularly within NHB.

## 1. Introduction

In 2020, breast cancer (BC) was the primary driver of global cancer incidence, accounting for an estimated 2.3 million new cases (11.7% of all cancer cases) [1]. In the United States (US), projections for 2023 indicate an estimated 300,590 new BC cases and 43,700 BC-related fatalities [2]. BC is the most prevalent form of cancer worldwide, with around 91% of individuals diagnosed with BC achieving a minimum five-year survival rate [1,3]. However, for every molecular subtype and stage of disease (except stage I), Black women exhibit the lowest 5-year relative survival rate compared to all other racial/ethnic groups [4]. The most significant disparities between Black and White women are observed in hormone-receptor-positive/human epidermal growth factor receptor 2-negative disease, with survival rates of 88% and 96% for Black and White women, respectively [4].

Cardiovascular disease (CVD) is the leading cause of death among patients with active BC over 50 [5]. The risk of CVD-related mortality is higher in post-menopausal female BC survivors than in individuals without a BC history [5]. Effective management of preexisting CVD risk factors, such as diabetes mellitus and hypertension, significantly influences the prognosis of older BC patients [6]. Social determinants of health (SDOH) are defined as “the conditions in which people are born, grow, work, live, and age, and the wider set of forces and systems shaping the conditions of daily life” by the World Health Organization, contributing significantly to the development of CVD risk factors, morbidity, and mortality, especially within marginalized communities [7,8]. SDOH, encompassing factors such as poverty, limited education, neighborhood disadvantage, racial residential segregation, discrimination, insufficient social support, and isolation, significantly influence both the stage at which BC is diagnosed and the subsequent survival outcomes [9].

Machine Learning (ML) models have been increasingly used as prediction tools due to their potential greater performance compared to traditional regression models, and their capacity to learn and deal with data with multiple structures, especially clinical data [10,11,12]. These models operate by receiving input data and employing mathematical optimization and statistical analysis techniques to predict outcomes [13]. A meta-analysis published in 2020 demonstrated that ML algorithms exhibit a high level of accuracy in predicting CVD outcomes [13].

According to recent evidence, ML models incorporating SDOH data improve the risk prediction of in-hospital mortality after hospitalization for Heart Failure (HF), particularly among Black adults [14]. The inclusion of SDOH data elevated the model’s classification index (C-index) from 0.72 (95% confidence interval [CI] 0.73–0.79) to 0.77 (95% CI 0.71–0.75) for Black patients, yet this effect was not observed in non-Black patients [14]. However, to our knowledge, there are no studies examining whether the inclusion of SDOH data enhances the prediction of cardiovascular events in patients with BC. We hypothesize that ML models incorporating SDOH data will outperform models without this integration in predicting major cardiac events (MACEs) in BC patients, especially in patients who are non-Hispanic Black (NHB). The primary objective of this study is to develop and compare race-specific (separate models for NHB and non-Hispanic White (NHW) patients) and race-agnostic (race as a covariate) ML models with and without SDOH data in the prediction of MACE in patients with BC.

## 2. Materials and Methods

### 2.1. Study Setting

The study setting was the University Hospitals (UH) Seidman Cancer Center in Northeast Ohio, US. UH is a large hybrid academic-community tertiary care center that provides medical services to diverse communities, including urban, suburban, and rural areas. It comprises an extensive network comprising 23 hospitals, over 50 health centers and outpatient facilities, and more than 200 physicians’ offices across 16 counties in the region [15,16]. The patient population at UH is predominantly from inner-city areas, leading to a higher representation of Black patients and comparatively lower percentages of Hispanic and Asian minorities than the US population [15,16].

### 2.2. Data Source

The data for this study were collected from the UH Seidman Cancer Center data repository, which is based on the CAISIS platform, an open-source, web-based cancer data management system that integrates multiple sources of patient data [16,17,18,19,20,21,22]. To enhance the accuracy and comprehensiveness of the obtained information for each patient, additional data from Electronic Health Records (EHR) were incorporated using the Electronic Medical Record Search Engine (EMERSE) [23]. All patient records were deidentified.

### 2.3. Inclusion and Exclusion Criteria

**Figure 1:**
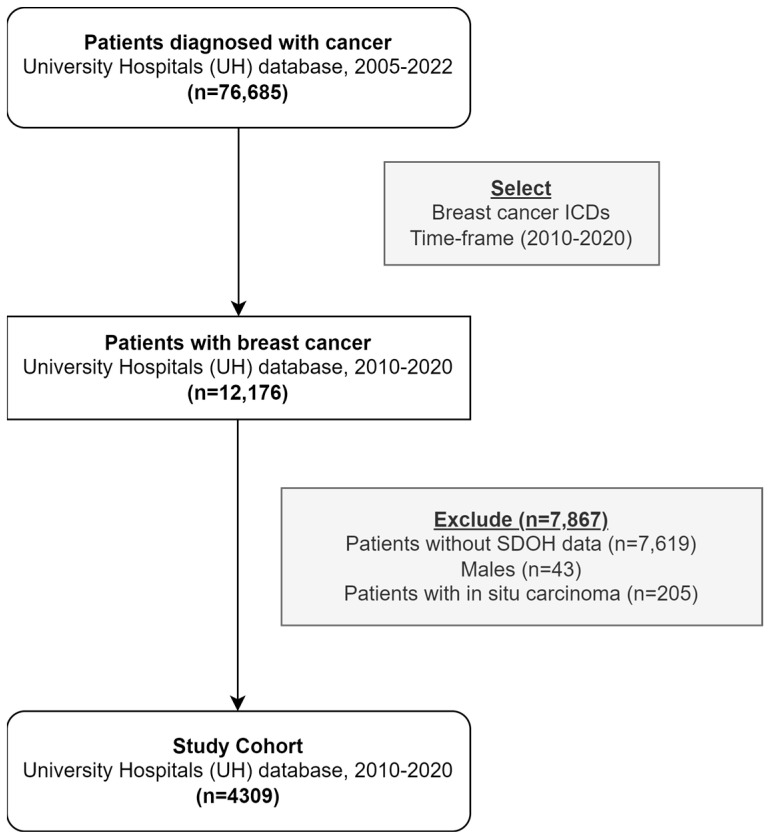
Study consort diagram for breast cancer patients, University Hospitals (UH) population (2010–2020).

The cohort (Figure 1) consisted of females aged 18 years or older diagnosed with BC at any stage. The diagnosis was determined based on specific ICD 9/10 codes, including C50.XX, C79.81, 174.X, 175.0, 175.9, 198.81, and 217, where “X” represents any integer [24,25]. The inclusion criteria encompassed patients diagnosed between 1 January 2010 and 31 December 2019, ensuring a minimum follow-up period of two years by the year 2022, which was the data collection year. Patients were excluded from the analysis if they were male or had in situ carcinoma. Due to a low number of patients with Hispanic ethnicity, these individuals were also excluded from the analysis. All patients with available SDOH data were included, while patients without SDOH data were excluded from the analysis.

### 2.4. Outcome

The co-primary outcomes of this study were the diagnosis and time-to-event occurrence of 2-year MACE following the diagnosis of BC. The MACE events considered included heart failure (HF), acute coronary syndrome (ACS), atrial fibrillation (A-fib), and ischemic stroke (IS) [16,26]. The diagnosis of these events was determined using specific ICD 9/10 codes obtained from the complete medical history recorded in the EHR of each patient.

### 2.5. Covariates

Data on demographics, risk factors, SDOH, tumor characteristics, and treatment were collected for all eligible patients. Demographic information obtained from the patient’s EHR included age at diagnosis, self-reported race/ethnicity (NHB, NHW, other), and payer information (Medicaid, Medicare, private insurance, self-pay, other). Risk factors were extracted from the comorbidities list using relevant ICD codes identified prior to the MACE diagnosis. These risk factors encompassed self-reported smoking status (yes, no, former, unknown), Charlson comorbidity index, and cardiovascular (CV) history/risk factors (yes, no) [27,28]. Positive CV history/risk factors were identified if the patient had a diagnosis of hyperlipidemia, cardiomyopathy, known coronary artery disease, prior myocardial infarction (MI), carotid disease, prior transient ischemic attack (TIA)/stroke, and/or chronic kidney disease (CKD) (Supplemental Table S1). Combining these factors into a single variable aimed to generate a covariate that characterizes patients at high CV risk [29].

Individual and neighborhood-level SDOH features were sourced from LexisNexis, the world’s largest electronic database for legal and public-records-related information. These features were grouped into four domains: social and community context (marital status, number of household members, distance to closest relatives), economic stability (address stability, property status, annual income, properties owned, wealth index, household income, total count of transport properties owned), neighborhood and built environment (crime index, burglary index, car theft index, murder index, neighborhood median household income, neighborhood median home values), and educational access and quality (education institution rating, college attendance) [30,31]. The LexisNexis dataset utilized in our study consists of a compilation of various public and private records that are updated at different frequencies, with the data obtained reflecting the most current available records and combining records from adult patients discharged from a UH facility over 2.5 years and adult patients who are members of an Accountable Care Organization [32].

Tumor characteristics included date of cancer diagnosis, hormone receptor status (estrogen receptor (ER), progesterone receptor (PR), and HER2), histological type (ductal or lobular, not specified (NOS), other/unknown), and TNM staging group (stage 0–IV). Treatment characteristics encompassed appointment completion rates and the use of single or combination treatments throughout a patient’s follow-up, including radiation of the breast (right, left), chemotherapy, endocrine therapy, and immunotherapy.

### 2.6. Descriptive Analysis

To ensure the integrity and reliability of our dataset for analysis, we implemented an outlier detection procedure [33]. This involved the application of data visualization techniques, specifically utilizing box plots, to effectively identify and subsequently remove outliers from the dataset [34].

The data were categorized based on race/ethnicity (NHB, NHW) and presented as absolute values and percentages for categorical variables and as median and quartiles for continuous variables. To compare categorical variables among different racial/ethnic groups, the Pearson chi-square test was employed. The distribution assumptions of continuous variables were assessed using histograms and the Kolmogorov–Smirnov test. Student’s *t*-tests were conducted for normally distributed factors, while non-parametric Kruskal–Wallis tests were used for non-normally distributed factors.

Correlation plots were used to examine the correlations among independent variables, and variables that exhibited statistically significant correlations were not included simultaneously in the models. A significance level of *p* < 0.05 was considered, and missing values were excluded from the analysis.

### 2.7. Machine Learning Development

**Figure 2:**
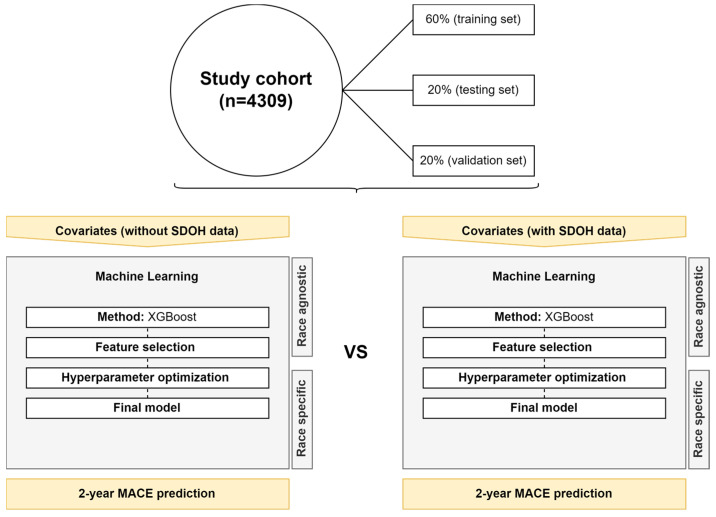
Study machine learning design detailing race-specific and race agnostic models.

Race-specific and race-agnostic ML models, with and without SDOH data, were developed and compared (Figure 2). The ML approach was chosen in this study due to its ability to learn from data and handle diverse data structures [14,35,36]. We utilized the tree-based method called extreme gradient boosting (XGBoost), designed for ML in survival analysis [37,38].

The preprocessing phase encompassed three main stages: data splitting, feature engineering, and feature selection. During the data split, the data were chronologically divided into three sets: 60% for training, 20% for testing, and 20% for validation [39]. In the process of feature engineering, categorical variable columns were transformed through transposition, resulting in the creation of individual binary classification columns for each category—in this new scheme, a value of 1 represented true, while 0 denoted false [40]. Feature selection was performed on the training set by comparing variables based on their association with MACE (yes vs. no), selecting those with a *p*-value less than 0.30, a conservative approach to avoid the exclusion of relevant covariates [41]. The testing set was used for hyperparameter tuning using a 10-fold 10-times cross-validation with 100 iterations, prioritizing the C-index [42]. All the models were adjusted for the following hyperparameters: nrounds (number of additional trees or weak learners added to the model), nthread (number of parallel threads used), eta (shrinkage of feature weights in each boosting step), max_depth (the maximum depth of each tree), min_child_weight (the minimum weight/number of samples required to create a new node in the tree), gamma (the minimum loss reduction to create new tree-split), subsample (the fraction of observations/rows to subsample at each step), and colsample_bytree (percentage of features/columns used to build each tree). The hyperparameter tuning was conducted using the randomized search approach [43]. Subsequently, the tuned model was applied to the validation set using a 10-fold, 10-times cross-validation. The performance of the ML models was assessed using the mean C-index, a precise and appropriate technique for measuring prediction error, along with its 95% CI [42,44,45]. The models ultimately chosen following the aforementioned phases were the ones exhibiting the highest C-index values.

### 2.8. Software and Packages

The analyses were conducted using RStudio software, version 4.2.2 [46]. The ML models were developed using the “mlr3” (version 0.16.1) and “mlr3proba” (version 0.5.2) packages [47,48].

## 3. Results

### 3.1. Population

We included 4309 females with BC (Figure 1; Table 1), of which 765 (17.8%) were categorized as NHB. The median age at diagnosis for the cohort was 63 years, with an interquartile range (IQR) of 53 to 72 years. Ductal carcinoma accounted for 49.2% of the diagnoses, while 5.7% were classified as stage III and 1.9% as stage IV. Among the cases, 44.9% were ER-positive, 40.2% were PR-positive, and 6.8% were HER2-positive. Most patients were never smokers (50.6%) and had a history or risk factor for cardiovascular disease (74.6%). The median Charlson comorbidity score was 4 (IQR 2–7). Surgery was performed in 60% of the cohort, while 28.2% received chemotherapy, 46% received endocrine therapy, 4.7% received immunotherapy, and 39.4% received radiotherapy.

### 3.2. Outcomes

Within a two-year follow-up period after the BC diagnosis, 11.4% of the patients experienced a MACE, with a median time-to-event of 177 days and an IQR of 45 to 414 days. HF was the most commonly diagnosed event, occurring in 6.9% of the patients, followed by A-fib in 3.7%, IS in 2.4%, and ACS in 2.3%. When comparing NHB individuals to NHW individuals, significantly higher rates of MACE (19.2% vs. 9.9%), HF (13.1% vs. 5.5%), and ACS (4.8% vs. 1.7%) were observed among NHB patients (*p* < 0.001). Moreover, NHB individuals had a rate of IS of 3.4% and A-fib of 3.8%, while NHW had rates of IS of 2.3% and A-fib of 3.8%. There were no notable differences in the time-to-event between racial/ethnic groups.

### 3.3. Race-Agnostic ML Models

The race-agnostic models with and without SDOH data were developed in 4309 female patients with BC (Table 2). The model without SDOH data exhibited a C-index of 0.78 (95% CI 0.76–0.79), while the model with SDOH data exhibited a C-index of 0.81 (95% CI 0.80–0.82).

### 3.4. Race-Specific ML Models—NHB

The race-specific models in NHB were developed in 765 patients (Table 2). The model without SDOH data exhibited a C-index of 0.74 (95% CI 0.72–0.76). The model with SDOH data exhibited a C-index of 0.75 (95% CI 0.73–0.78).

### 3.5. Race-Specific ML Model—NHW

The race-specific models in NHW were developed in 3321 patients (Table 2). The model without SDOH data exhibited a C-index of 0.79 (95% CI 0.77–0.80). The model with the SDOH data model exhibited a C-index of 0.79 (95% CI 0.77–0.80).

## 4. Discussion

This study aimed to develop and compare race-specific and race-agnostic ML models, with and without SDOH data, in predicting MACE in patients with BC. Our findings indicate that including SDOH data significantly improved the predictive performance of the ML models in NHB patients. Conversely, for NHW patients, the addition of SDOH data did not result in a noticeable change in the model’s performance, suggesting that other factors may have a more prominent role in driving MACE development in this group. Racial disparities in SDOH may contribute to the higher incidence of MACE in NHB patients, further emphasizing the social construct of race.

As a field, cardiology has been at the forefront of adopting ML techniques [49,50,51,52]. Several studies have demonstrated that ML algorithms outperform traditional risk assessments that rely on established CVD risk factors [13,53,54]. Conventional CVD risk assessment models often assume a linear relationship between each risk factor and CVD outcomes [55]. In addition, these models have limitations, including variations among specific populations, the overestimation of CVD risk in certain situations, and a limited number of predictors [56,57]. In previously published ML models for CVD prediction that did not incorporate SDOH data, most shared a common set of demographic variables (e.g., age, sex, smoking status) and laboratory values [13]. Our results encourage the integration of SDOH into ML algorithms developed for predicting CVD in patients with BC.

Traditional clinical risk factors for CVD have long been acknowledged in prevention efforts [58]. However, there is increasing recognition of the significant role played by the SDOH in the development of CVD [7]. Recent evidence has shown that specific SDOH, such as socioeconomic status (SES), race and ethnicity, social support, cultural and language factors, access to healthcare, and residential environment, play a crucial role in predicting disparities in CVD risk and CVD outcomes [7]. A lower SES is hypothesized to act as a chronic stressor, contributing to promoting a proinflammatory state and developing atherosclerosis [59,60,61,62]. The chronic stress associated with lower SES can be quantified using allostatic load, which is linked to a significant increase of up to 31% in CVD risk [21]. Taking into account the aspect of the neighborhood-built environment (which refers to the physical characteristics and design of neighborhoods), research has consistently demonstrated that adverse neighborhood conditions such as higher population density; increased traffic; limited availability of nearby stores, supermarkets, and fitness centers; and insufficient green spaces or vegetation are associated with an elevated CVD risk [63,64,65,66,67]. Furthermore, psychosocial factors (psychological and social characteristics) play a crucial role in CVD—various factors within this domain, including job strain, childhood experiences, depression, perceived discrimination, and social isolation, have been shown to have significant associations with the development and progression of CVD [68,69,70,71,72,73,74,75,76]. Our findings reaffirm the crucial role of SDOH in CVD. We observed a noteworthy enhancement in the predictive performance of the race-agnostic model when incorporating SDOH data, with the model’s C-index increasing from 0.78 to 0.81. This underscores the significance of considering SDOH factors in improving the accuracy of CVD prediction models.

Notably, our results have shown that the predictive performance after including SDOH data is higher in NHB compared to NHW. This highlights the importance of understanding racial disparities and conceptualizing race as a social construct. Structural racism can contribute to residential segregation, which in turn influences employment prospects, economic status, access to quality education, and exposure to higher levels of neighborhood violence, crime, and poverty [7]. An illustrative example of this effect is the higher likelihood of Black individuals residing in states with high levels of structural racism reporting a history of MI within the past year compared to Black individuals in states with low levels of structural racism [7]. Focusing specifically on patients with BC, it is hypothesized that adverse SDOH may explain the racial disparities observed in CVD outcomes within this population, as NHB women with BC face greater adversity in SDOH factors [16]. This is of utmost importance considering the higher MACE/CVD rates observed in NHB individuals, as confirmed by our study results [16].

From a practical standpoint, the findings of our study align with the principles outlined in the 2023 American Heart Association statement titled “Equity in Cardio-Oncology Care and Research”, emphasizing the need to implement strategies that mitigate inequalities and address the healthcare needs of underserved populations [77]. The results underscore the urgency of developing public health policies aimed at addressing disparities in SDOH. Immediate action is needed to ensure equitable healthcare access and tackle the underlying factors contributing to SDOH disparities. Furthermore, our study has demonstrated the importance of integrating SDOH data into future predictive models to enhance their performance.

This study possesses several limitations. First, the database used in this study relies on EHR, and some information may be incomplete or missing. Furthermore, while our institution maintains a close follow-up with patients as a nationally recognized comprehensive cancer center, some patients may still be lost to follow-up or seek emergency care at other healthcare facilities, which could introduce a potential bias. Additionally, the criteria for data availability in LexisNexis may have led to a selection bias in our sample. The results reported may reflect the characteristics and demographics of the catchment area where our institution is located and may represent individuals with a higher propensity for seeking healthcare services. Moreover, including both patients with curable and incurable BC could have influenced the reported rates of MACE. The ML models were not validated in an external dataset.

## 5. Conclusions

In summary, there is an improvement in the predictive performance of machine learning models for predicting MACEs in patients with BC with the incorporation of social determinants of health (SDOH) data, particularly NHB patients. These findings underscore that race is a social construct and emphasize the importance of public policies to reduce inequalities and address SDOH disparities. Future studies should consider prospective and multicenter designs or US nationally representative samples, encompass diverse populations, explore a broader range of covariates, develop specific models for different types of CVD, scrutinize optimal cut-off points for individual models, and investigate the geographical variations in SDOH within regions.

## Figures and Tables

**Table 1:** Population characteristics from patients with breast cancer at University Hospitals (UH) Seidman Cancer Center, 2010–2020.

	Patients Diagnosed with Breast Cancer
	University Hospitals (UH), 2010–2020
	*n* = 4309
Age at diagnosis—median (IQR)	63 (53–72)
Race/ethnicity—*n* (%)	
non-Hispanic Black	765 (17.7)
non-Hispanic White	3321 (77.1)
Other	223 (5.2)
Stage—*n* (%)	
III–IV	326 (7.5)
Histology—*n* (%)	
Ductal	2121 (49.2)
ER+—*n* (%)	1936 (44.9)
PR+—*n* (%)	1732 (40.2)
HER2+—*n* (%)	90 (2.1)
Smoking status—*n* (%)	
Smoker	303 (7)
Former smoker	9897 (22.9)
Never smoker	2182 (50.6)
Unknown	837 (19.4)
Charlson comorbidity score—median (IQR)	4 (2–7)
Cardiovascular history/risk factor—*n* (%)	3123 (74.6)
Cardiomyopathy	230 (5.3)
Coronary artery disease (CAD)	775 (18)
Myocardial infarction (MI)	261 (6.1)
Carotid disease (CD)	141 (3.3)
Transient ischemic attack (TIA)/Stroke	67 (1.6)
Chronic kidney disease (CKD)	536 (12.4)
Dyslipidemia	2285 (5.3)
Diagnosis per patient—median (IQR)	2 (0–2)
Surgery—*n* (%)	
Mastectomy	792 (18.4)
Lumpectomy	964 (22.4)
Chemotherapy (C)—*n* (%)	1213 (28.2)
Radiotherapy (R)—*n* (%)	1699 (39.4)
Left	401 (9.3)
Right	436 (10.1)
Immunotherapy (I)—*n* (%)	204 (4.7)
Endocrine therapy (E)—*n* (%)	1982 (46)
Combined therapy—*n* (%)	
C + R	761 (17.7)
I + R	123 (2.9)
H + C + R	459 (10.7)
H + C + R + I	61 (1.4)
% appointments attended—median (IQR)	66.6 (50–81.8)

**Table 2:** Hyperparameters and performance for race-agnostic and race-specific ML models designed to predict 2-year MACE.

		Hyperparameters	Performance (C-Index)
Race-agnostic	Without SDOH data	nrounds = 2050; nthread = 10; verbose = 0; eta = 0.02715107; max_depth = 9; min_child_weight = 2.886243; gamma = 3.93808; subsample = 0.9668632; colsample_bytree = 0.9550104	0.78 (0.76–0.79)
	With SDOH data	nrounds = 50; nthread = 8; verbose = 0; eta = 0.1013887; max_depth = 1; min_child_weight = 2.971928; gamma = 3.337559; subsample = 0.804832; colsample_bytree = 0.97875	0.81 (0.80–0.82)
NHB	Without SDOH data	nrounds = 50; nthread = 14; verbose = 0; eta = 0.02364827; max_depth = 1; min_child_weight = 2.62171; gamma = 4.533674; subsample = 0.9894932; colsample_bytree = 0.6737331	0.74 (0.72–0.76)
	With SDOH data	nrounds = 50; nthread = 16; verbose = 0; eta = 0.04240374; max_depth = 4; min_child_weight = 7.789127; gamma = 4.256919; subsample = 0.9581859; colsample_bytree = 0.6278961	0.75 (0.73–0.78)
NHW	Without SDOH data	nrounds = 50; nthread = 4; verbose = 0; eta = 0.03734001; max_depth = 2; min_child_weight = 2.380759; gamma = 4.503645; subsample = 0.8980231; colsample_bytree = 0.8306106	0.79 (0.77–0.80)
	With SDOH data	nrounds = 4050; nthread = 14; verbose = 0; eta = 0.06144029; max_depth = 2; min_child_weight = 0.1104873; gamma = 2.937595; subsample = 0.999557; colsample_bytree = 0.8240068	0.79 (0.77–0.80)

## Data Availability

University Hospitals (UH) Seidman Cancer Center database is available at UH Cleveland Medical Center and has access restricted to researchers with IRB approval.

## Supplementary Materials

The following supporting information can be downloaded at https://www.mdpi.com/article/10.3390/cancers15184630/s1, Table S1: ICD codes used to categorize cardiovascular history/risk factors and MACE. Medications names included on each medication category. Social determinants of health variables from LexisNexis and its domain categorization.

## Abbreviations

ACS	acute coronary syndrome
A-fib	atrial fibrillation
BC	breast cancer
CI	confidence interval
CVD	cardiovascular disease
CV	cardiovascular
C-index	concordance index
EMERSE	Electronic Medical Record Search Engine
ER	estrogen receptor
HER	eletronic health records
HF	heart failure
IQR	Interquartile range
ICD	International Classification of Diseases
IS	ischemic stroke
MACE	major cardiac events
ML	machine learning
MI	myocardial infarction
NHB	non-Hispanic Black
NHW	non-Hispanic White
NOS	not specified
PR	progesterone receptor
SDOH	social determinants of health
SES	socioeconomic status
TIA	transient ischemic attack
US	United States
UH	University Hospitals
