# Supplementary material for: Social Determinants of Health Data Improve the Prediction of Cardiac Outcomes in Females with Breast Cancer
Source: Cancers (Basel). 2023 Sep 19;15(18):4630. doi: 10.3390/cancers15184630 (PMC10526347; doi:10.3390/cancers15184630)
Supplement: Supplementary file 1 [file cancers-15-04630-s001.zip › cancers-2559827-supplementary.pdf]

Supplemental Material

Title: Social Determinants of Health Data Improve the Prediction of Cardiac Outcomes in Females with Breast Cancer

Supplemental Table S1. ICD codes used to categorize cardiovascular history/risk factors and MACE. Medications names included on each medication category. Social determinants of health variables from LexisNexis and its domain categorization.

**Cardiovascular risk factors**	**ICD9**	**ICD10**
Cardiomyopathy	425.XX	I42.XX
Known Coronary Artery Disease	414.00, 414.01, 414.02, 414.03, 414.04, 414.05, 414.06, 414.07	I25.10, I25.810, I25.811, I25.812
Prior Myocardial Infarction	412.XX	I25.2
Carotid Disease	433.10	I65.29
Prior TIA/Stroke	438.XX	I69.9XX
Chronic Kidney Disease	585.XX	N18X
Dyslipidemia	272.X	E78X
**MACE**	**ICD9**	**ICD10**
Heart Failure	428.XX, 398.9, 402.XX, 401.XX, 414.8, 425.XX,785.51	I50.XX, I09.9, I11.0, I13.0, I13.2, I25.2, I42.0, I42.5-I42.9, I43.XX, R570
Ischemic Stroke	431.XX, 435.0X, 435.1X, 435.2X,<br>435.3X, 435.8X, 435.9X, 433.01,<br>433.11, 433.21, 433.31, 433.81,<br>433.91, 434.01, 434.11, 434.91,<br>997.01, 344.60, 344.61	I619, G45X, I63, I66, G834, G9781
Myocardial Infarction	411.XX, 410.XX	I20.0, I21.X, I24.X
Atrial Fibrillation	427.31	I48.91
**SDOH variable**	**Description**	
Domain 1: Social and Community Context		
Marital Status	Individual's inferred marital status	
Number of household members	Total count of household members who are on record	
Number of elderly household members	Total count of household members who are on record age 80 and older	
Number of middle age household members	Total count of household members who are on record age 40 to 64	
Number of young adult household members	Total count of household members who are on record age 20 to 39	

Closest relatives distance	Distance in miles between identity and closest first degree relative or close associate
Domain 2: Neighborhood and Built Environment	
Neighborhood burglary index	Current address neighborhood burglary index based on FBI data
Neighborhood car theft index	Current address neighborhood motor vehicle theft index based on FBI data
Negihborhood crime index	Current address neighborhood total crime index based on FBI data
Neighborhood median household income	Current address neighborhood median household income based on US Census data
Neighborhood murder index	Current address neighborhood murder index based on FBI data
Neighborhood median home value	Input address neighborhood median home values based on US Census data
Domain 3: Education Access and Quality	
Attended college	Indicates that the individual is reported as having attended college
Attending college	Indicates that the individual is reported as currently attending college
Education institution rating	Indicates the education program rating based on various institution statistics and national ratings
Domain 4: Economic Stability	
Annual income	Identity's estimated annual income rounded to the nearest $1000
Owns a property	Indicates if identity is associated with ownership of any real property
Wealth Index	Wealth index based on relative value of real property and other assets recently reported
Number of properties owned	Number of properties identity currently owns
Household income	Estimated household income range, based on property value records and asset records that are associated with household members
Number of transportation properties in the household	Total count of personal property registrations (automobile, motorcycle, watercraft, or aircraft) that are currently registered to household members who are on record, based on personal property ownership records
Address stability index	Address stability index indicating how often identity has changed addresses
Current residence status	Indicates if identity owns or rents at current address
